# Mortality From Alcohol-Related Liver Cirrhosis in Mexico (2000–2017)

**DOI:** 10.3389/fpubh.2020.524356

**Published:** 2020-10-23

**Authors:** Myrna L. Yeverino-Gutiérrez, María del R. González-González, Omar González-Santiago

**Affiliations:** ^1^Laboratory of Pharmacology and Toxicology, School of Chemical Science, Universidad Autonoma de Nuevo Leon, San Nicolás de los Garza, Mexico; ^2^Pharmacy, School of Chemical Science, Universidad Autonoma de Nuevo Leon, San Nicolás de los Garza, Mexico

**Keywords:** cirrhosis, mortality, Mexico, liver, alcohol

## Abstract

**Background:** Alcohol is the main cause of liver cirrhosis. The objective of this study was to analyze the mortality rates of alcohol-related cirrhosis in Mexico from 2000 to 2017.

**Methods:** Mortality data from alcohol-related cirrhosis were obtained from the National Institute of Statistics and Geography. Rates were adjusted to the World Standard Population and were calculated with a direct method. The differences between genders were evaluated with Student's *t-*test, while the ANOVA test was used for differences among age groups. A trend analysis was performed with an ln regression of adjusted mortality rates and analyzed with Student's *t*-test.

**Results:** The mean age-adjusted mortality rate during the study period was 13.28 per 100,000 inhabitants. A significant decrease in mortality rates was observed, from 20.55 to 10.62 per 100,000 inhabitants. All age groups studied showed a significant decrease in mortality. The mortality rate was higher in males than in females.

**Conclusions:** Mortality from alcohol-related cirrhosis decreased in Mexico. Males still have the highest mortality rate.

## Introduction

The degree of alcohol consumption and its relationship to health is a well-known topic. Excessive alcohol consumption increases the risk of heart disease and stroke (1) in addition to being an important cause of cirrhosis (2). The World Health Organization estimated in 2018 that 48% of all cirrhosis-related deaths worldwide were attributable to chronic alcohol consumption. The same publication reported that alcohol-related liver cirrhosis deaths were higher than diabetes, AIDS, and tuberculosis (3).

Cirrhosis is the final stage of chronic liver disease that has a variety of causes, such as viral hepatitis, excessive alcohol consumption, non-alcoholic steatohepatitis, autoimmune liver disease, and genetic disorders, among others. These illnesses distort the hepatic architecture by fibrosis and cause the subsequent formation of regenerative nodules (4). The complications of cirrhosis include the development of ascites, hepatic encephalopathy, bleeding from gastroesophageal varices, cancer, and, in general terms, a reduction in life expectancy. When decompensation develops, a liver transplant is the only treatment that can save a life. The main mechanisms by which alcohol intake causes hepatocellular damage are oxidative stress, production of endotoxins, and abnormal methionine metabolism (5).

According to the Global Burden of Disease study 2017, liver cirrhosis caused 41.4 million Disability Adjusted Life Years (the years of life lost due to pre-mature death or healthy life lost due to disability), 12 million in females and 28 million in males (6). Cirrhosis caused 1.32 million deaths worldwide in the same year, which corresponds to 2.4% of all deaths (7). On the other hand, in Mexico, cirrhosis represents the fourth leading cause of death, with this being the highest in Latin America (8). Alcohol intake remains the most common cause of cirrhosis, and alcohol-related cirrhosis has been reported as the main cause of death due to liver disease in Mexico. According to projection studies, mortality due to cirrhosis will increase in the future (9).

Considering that alcohol is the main risk factor for the development of liver cirrhosis and due to the scarcity of reports in our country, the objective of this study was to describe the age-standardized mortality rate of alcohol-related cirrhosis in Mexico from 2010 to 2017.

## Methods

### Data

Population data from the years 2000 (10), 2010 (11), and 2015 (12) were obtained from the respective census reported by the National Institute of Statistics and Geography (INEGI, for its acronym in Spanish). For the remaining years, the population was estimated by linear interpolation and extrapolation. Deaths from alcoholic cirrhosis were grouped by gender into three age groups (<40, 40–60, and >60 years old); this as a way to represent young adult, middle adult, and elderly. Mortality data from alcohol-related cirrhosis were obtained from the INEGI. In this database, all deaths (100%) with a death certificate are registered. The codes used were K70.3—alcoholic cirrhosis of liver, and K70.4—alcoholic hepatic failure, of the International Classification of Disease 10. Raw data are available as a Supplementary Material.

### Statistical Analysis

Mortality rates were calculated and standardized using the World Standard Population. Crude mortality rates were calculated dividing the number of deaths due to alcohol-related cirrhosis by the expected population from the whole country for the respective year. Crude mortality rates were expressed per 100,000 inhabitants. The adjusted mortality rates were subsequently calculated with a direct method, multiplying each of the age-specific crude rates by the proportion of the World Standard Population (13) belonging to that particular age group. This produces direct age-standardized mortality rates that a country would have if they had the same age distribution as the standard population. To obtain adjusted rates for each gender and age group, we apply the same procedure, calculate crude rates, and multiply these crude rates by the proportion of the world standard population of each group. The differences between genders were evaluated with Student's *t*-test while an ANOVA was used to test the differences between age groups. The mortality trend was evaluated with a log regression of adjusted rates, where the independent variable was the year. The slope equal to zero was the null hypothesis, and it was tested with Student's *t*-test. The annual percent change (APC) was calculated with the following equation: APC = (e^m^ – 1)^*^100, where e raised to the m is the anti-natural logarithm (ln) of the slope of the ln regression rates. The statistical analysis software NCSS version 12 was used for data analysis. Tests were considered significant at *p*-value < 0.01. Since this study is retrospective and data are freely available, ethics committee approval was not necessary.

## Results

In Mexico, during the study period 2000–2017, the INEGI reported almost 10 million deaths; of these deaths, 235,690 (2.4%) were due to alcohol-related liver cirrhosis. According to the number of deaths due to alcohol-related cirrhosis, there were 8.3 times more deaths in males than in females, 210,536 and 25,100, respectively. According to the adjusted rates, male mortality was 9.2 times higher than female, 24.95, and 2.70 per 100,000 inhabitants, respectively (Table 1).

**Table 1:** Mortality rate from alcohol-related cirrhosis in Mexico.

**Group**	**Average 2000–2017**	**Standard deviation**
Total	13.28	3.14
**Gender**
Male	24.95	5.43
Female	2.70	1.01
*p*-value	<0.01	
**Both genders age group, years**
<40	2.65	0.57
40–60	32.18	7.75
>60	50.55	9.73
*p*-value	<0.01	
**Male age group, years**
<40	5.14	1.04
40–60	60.50	13.14
>60	94.50	16.48
*p*-value	<0.01	
**Female age group, years**
<40	0.4	0.16
40–60	6.35	2.51
>60	12.22	3.58
*p*-value	<0.01	

*Rates are per 100,000 habitants*.

**Figure 1:**
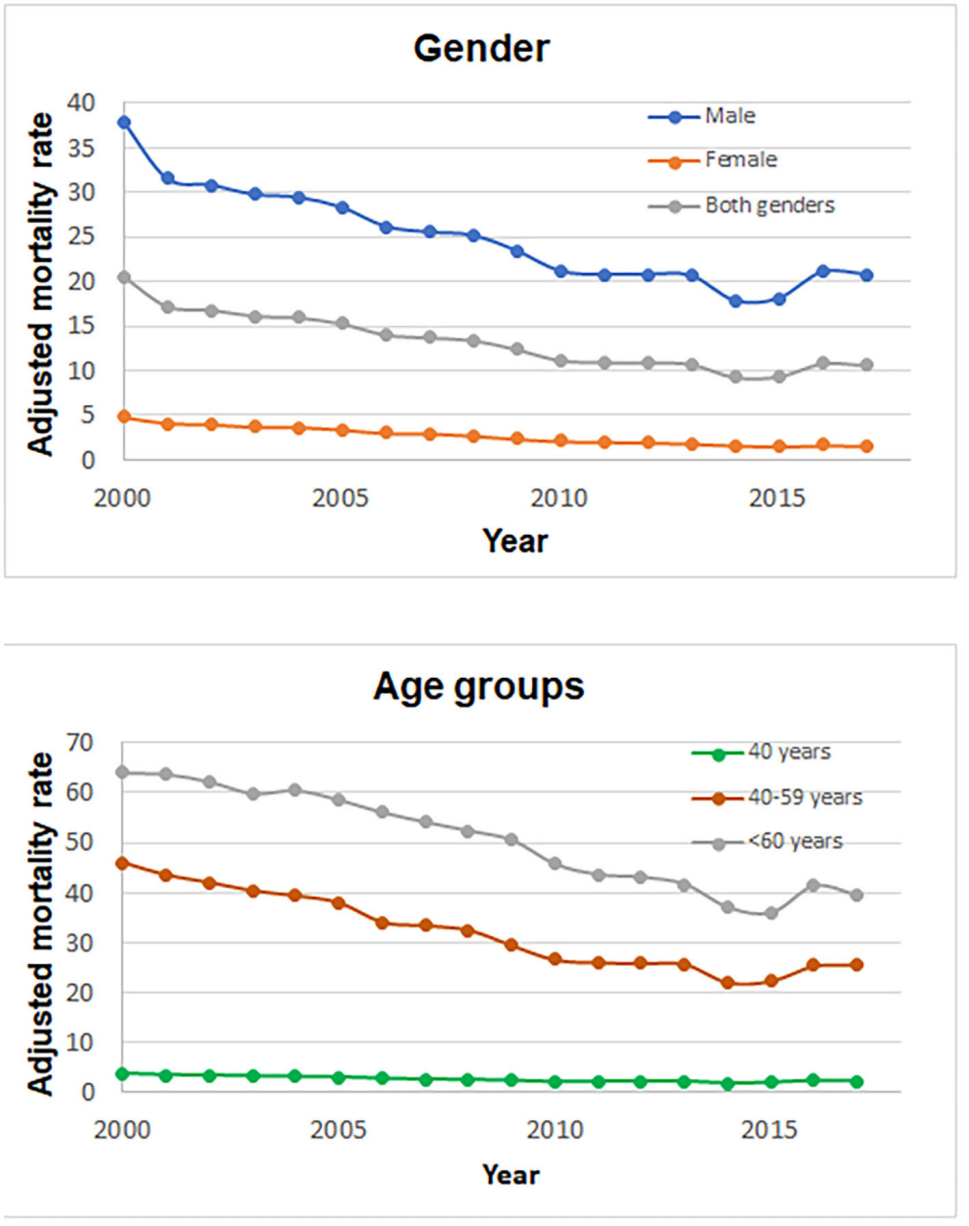
Mortality rate from alcohol-related cirrhosis in Mexico during 2000–2017 according gender and age group.

Between 2000 and 2017, the age-adjusted mortality rate decreased in both genders from 37.92 to 20.80 per 100,000 inhabitants in the case of males and from 4.75 to 1.57 per 100,000 inhabitants in the case of females (Figure 1).

The average age-adjusted mortality decreased during the period 2000–2014 from 20.55 to 9.28 per 100,000 inhabitants; however, a change in trend was observed in 2015. In that year, the mortality rate was 9.3, while in 2016 and 2017, it was 10.79 and 10.62 per 100,000 inhabitants respectively. On average, the mortality rate during the entire study period was 13.28 per 100,000 inhabitants (SD = 3.14) (Table 1). A significant difference was observed among the age groups of both males and females (*p* < 0.01) (Table 1).

The age-adjusted mortality in the <40-year-old group (young adults) was 2.65 per 100,000 habitants (SD = 0.65); in the 40- to 60-year-old group, it was 32.18 per 100,000 habitants (SD = 7.75). In the >60-year-old group, the age-adjusted mortality rate was the highest with a rate of 50.55 (63.78–39.34) per 100,000 habitants (Table 1, Figure 1).

Annual percent change analysis showed a significant decrease in the mortality of all the groups studied; in females, a higher decrease in mortality rates than males was observed (−6.70 vs. −3.64%). On the other hand, the 40- to 60-year-old group showed the highest decrease in mortality (−4.19%) compared to the rest of the groups in both genders (Table 2).

**Table 2:** Average annual percentage change in alcohol related cirrhosis, 2000–2017.

**Group**	APC^*^	**Test for trend**
Total	−4.02	<0.01
**Age group, years**
<40	−3.56	<0.01
40–60	−4.19	<0.01
>60	−3.51	<0.01
Male	−3.64	<0.01
**Age group, years**
<40	−3.28	<0.01
40–60	−3.75	<0.01
>60	−3.15	<0.01
Female	−6.7	<0.01
**Age group, years**
<40	−6.73	<0.01
40–60	−7.19	<0.01
>60	−5.42	<0.01

* *APC = Annual percentage change*.

## Discussion

In this short report, we describe trends in mortality due to alcohol-related cirrhosis. Our results show that the epidemiology of cirrhosis in Mexico changed during the study period. Data from OECD (14) showed that Mexico presented a decrease in alcohol consumption during the period from 1992 to 2012. This could explain the lower mortality rate and decreased trends observed in our study.

In European countries, alcohol-related cirrhosis mortality was very different from 1996 to 2004, with an average of 16.0 per 100,000 inhabitants (interquartile range = 9.6–35.2). Countries with rates higher than those observed in our study were Finland, Denmark, England, Switzerland, Slovenia, Hungary, Czech Republic, Lithuania, and Estonia. On the other hand, countries with lower rates than those observed in our study were Sweden, Norway, Austria, and Poland (15).

Contrary to our results, the United States had an increase in mortality trends due to alcohol-related cirrhosis from 4.3 to 5.8 per 100,000 inhabitants during 2000–2015 (16). The same occurred in Estonia during 1992–2008. Mortality rates increased in both genders: males from 3.4 to 43.6 per 100,000 inhabitants and females from 0.5 to 17.3 per 100,000 inhabitants. The authors conclude that this increase is due to an increase in alcohol consumption (17).

According to a Mexican survey, the Encuesta Nacional de Consumo de Drogas (National Survey of Drug Use) 2016–2017, alcohol consumption began to increase in Mexico from 2011, especially in young women (18). This could explain the slight increase in mortality in 2015.

In Mexico, more health policies are necessary, particularly those related to obesity and alcohol consumption, to avoid the most important causes of liver disease (8). Also, studies that analyze the magnitude of the effect that these policies could have on alcohol consumption are needed.

If the recent increase in alcohol consumption in Mexico is not reversed, an increase in the alcohol-related cirrhosis mortality trend will probably be consolidated in the short or medium term. In addition to this concern, the increase in alcohol consumption could have other serious implications in several sectors, such as public health (consultations, medicines, transplants, and pre-mature deaths) and the economy, (productivity, disabilities, and absenteeism).

In addition to changes in alcohol consumption, other causes that could explain the differences in the rates and trends of mortality due to alcohol-related cirrhosis in Mexico are not clear; therefore, more studies are needed.

Our study has some limitations because in Mexico there are very few publications concerning trends in alcohol consumption and alcohol-related morbidity and mortality. Other limitations are that there are no data about the incidence of cirrhosis in Mexico, as well as the severity of alcohol-related cirrhosis deaths.

In conclusion, alcohol-related cirrhosis mortality decreased in Mexico from 2000 to 2017. Males and the >60-year-old group presented the highest mortality.

## Data Availability Statement

All datasets generated for this study are included in the article/Supplementary Material.

## Author Contributions

OG-S and MY-G: designed this study. MG-G and MY-G: collected data and performed analysis. OG-S and MY-G: wrote the manuscript. OG-S, MY-G, and MG-G: reviewed the manuscript. All authors contributed to the article and approved the submitted version.

## Conflict of Interest

The authors declare that the research was conducted in the absence of any commercial or financial relationships that could be considered a potential conflict of interest.
